# Design of the Squared Daisy: A Multi-Mode Energy Harvester, with Reduced Variability and a Non-Linear Frequency Response

**DOI:** 10.3390/s19153247

**Published:** 2019-07-24

**Authors:** Mathieu Gratuze, Abdul Hafiz Alameh, Frederic Nabki

**Affiliations:** Department of Electrical Engineering, École de Technologie Supérieure, Montréal, QC H3C 1K3, Canada

**Keywords:** MEMS, energy harvesters, piezo-electricity, vibrations, aluminum nitride, process variation, non-linear, multi-mode

## Abstract

With the rise of the Internet of Things (IoT) and the ever-increasing number of integrated sensors, the question of powering these devices represents an additional challenge. The traditional approach is to use a battery; however, harvesting energy from the environment seems to be the most practical approach. To that end, the use of piezoelectric MEMS energy has been proven as a potential power source in a wide range of applications. In this work, a proof of concept for a new architecture for MEMS energy harvesters is presented. The influence of the dimensions and different characteristics of these designs is discussed. These designs have been proven to be resilient to process variation thanks to their unique architecture. This work presents the use of vibration enhancement petals in order to widen the bandwidth of the energy harvester and provide a non-linear frequency response. The use of these vibration enhancement petals has allowed the fabrication of three design variations, each using an area of 1700 µm by 1700 µm. These designs have an operating bandwidth between 3.9 kHz and 14.5 kHz and can be scaled to achieve other targeted resonant frequencies.

## 1. Introduction

Energy recovery systems and energy harvesting systems are the subjects of growing interest [1,2,3,4,5]. In addition, the number of connected objects is constantly increasing [6], and the energy requirements of these systems are still growing and many sensors are manufactured without it being physically possible or economically viable to replace their batteries once they are depleted [5]. The development of energy recovery and energy harvesting systems makes it possible to increase the lifespan of these objects, which is important notably in devices placed in remote locations or harsh environments.

Research and development of such energy harvesting systems has been focused on exploring the use of several different energy sources that can be converted into electrical energy. These energy sources need to be readily available in the environment. These include heat, light, magnetism, wind, and vibrations [7,8]. Several systems have been proposed to exploit one or more of these energy sources to produce electrical energy [9,10,11]. 

In this work, the use of vibrations as an energy source is considered by specifically focusing on piezoelectric energy harvesters. End applications include but are not limited to devices for biomedical, automotive, industrial or even military application [12,13,14]. When using vibrations as the main energy source, the device should be able to power the circuit in which it is integrated. Due to the nature of vibrations present in various environments, these devices need to have a low resonant frequency, and provide a sufficient power output while having a wide enough bandwidth to allow powering of the device over a wide range of vibration frequencies. These devices also need to be compact in order to allow their integrations into actual systems. However, achieving one of these objectives generally results in a challenge in maintaining the others. Therefore, a tradeoff has to be determined between the size of the device, its main resonant frequency, bandwidth, and maximum output power. Thus, the development of new structures for the implementation of piezoelectric vibration energy harvesters (PVEH) is necessary. To this end, this work will propose PVEH structures fabricated in silicon on an insulator microelectromechanical system (MEMS) fabrication process.

The most basic structure used for the realization of a MEMS PVEH is the cantilever [2,15,16,17,18]. The performance of such harvesters has been studied for energy harvesting purposes, and many different analytical models have been developed to explain and predict the behavior of this type of device. While these structures are relatively easy to fabricate and their behavior are well predicted, they are still the subject of research to overcome their limitations. Namely, the scaling down of the size of these devices proves to be a complex challenge, as their resonant frequency increases linearly with the scaling factor [19]. In addition, the smaller the device, the lower the output power.

In previous work, it has been explained how tuning the dimensions of a cantilever to attain a T-shape will allow for the reduction of up to 30% of the resonant frequency, however, this comes at the cost of reducing the power output [20]. It also has been theorized that linking four beams with a central proof mass in a cross design could be a step toward more efficient design than the cantilevers [21]. 

While it is possible to scale down and even tune the resonant frequency of these cantilevers by scaling them up, changing the material used for their realization, changing their geometry, using planar masses or even adding a proof mass [20], tuning the bandwidth of these designs is more difficult. While modifying the ambient pressure at which the device operates can provide such tuning, it is not practical and costly to package. The bandwidth of an energy harvester determines both its maximum output power and the frequency range in which the device can operate. This bandwidth is linked to the quality (Q) factor, and the higher the Q factor, the higher the maximum power output but the narrower the bandwidth. On the contrary, the lower the Q factor, the lower the maximum power output but the wider the bandwidth [8,14,22,23]. A trade-off in terms of the Q factor for the devices thus has to be realized in order to allow for both a sufficient output power while maintaining a wide enough bandwidth to accommodate a wide range of vibration frequencies. To overcome this trade-off, researchers have explored the design of devices using the coupling of two or more structures each with different resonant frequencies in order to allow for a wider bandwidth without compromising the maximum output power. 

As such, work in [16] presents a structure allowing a maximum power output of 20 µW, however, the proposed device is large (15 mm × 15 mm), and the resonant modes of the structure are spaced between 100 Hz and 1 kHz. Furthermore, is not possible to fabricate the device using MEMS technology, limiting the miniaturization potential of the design and its batch fabrication. To reduce the resonant frequency, the design in [17] utilizes the coupling of two cantilever beams to achieve a resonant frequency of 16 Hz with a bandwidth of about 14 Hz, and a maximum power output of 0.35 µW. To achieve such performance, this work required a relatively large device (55 mm × 18 mm). Using the same concept of using several coupled cantilevers, work in [2] presents another structure with an operating frequency between 10 Hz and 20 Hz. However, once again requiring large dimensions for the devices (90 mm × 10 mm), but achieving a maximum power output of 249.8 µW. Other strategies for wideband mechanical energy harvesting are detailed in [18]. A design proposed in [18] is a cantilever occupying an area of 30 mm × 10 mm with a frequency tuning system in the range of 210 Hz to 280 Hz. Work in [24] presents the use of a tri-legged structure to harvest random vibrations and produce up to 22 V, however once again the dimensions of the structure are relatively large (diameter of 52 mm). To add to the limitations of these devices, they cannot be integrated into compact embedded systems and batch fabricated at low cost, as none of these devices were fabricated using MEMS microfabrication technology. 

More compact devices using MEMS microfabrication technology are discussed below. Structures such as the one described in [1] have been reported with five resonances and 478 Hz of bandwidth, while keeping the dimensions relatively small (12 mm × 12 mm) and reporting a maximum output power of 1.73 µW. The scaling down of this structure proves to be detrimental, as illustrated by [25]. In that work, a similar structure is proposed as a vibration sensor and scaled to 2.35 mm × 2.35 mm with resonant frequencies of 14.53 kHz, 22.80 kHz and 22.81 kHz As this device is used as a vibration sensor, no output power is presented, but the work reports a sensitivity of 46 mV/g. Works in [13] and [3] present the Four-Leaf Clover, a structure with several resonances ranging from 200 Hz to several kHz and capable of delivering according to simulations a maximum power output of 11 µW at 230 Hz with dimensions of 8 mm × 8 mm. The work in [26] presents the use of structures with two resonant frequencies close to one another in order to present a much wider bandwidth. Simulations report a maximum of 10.1 µW and 9.3 µW at frequencies of 2.49 kHz and 2.55 kHz, occupying an area of 12.8 mm × 4.8 mm. Work in [5] presents the use of coupled cantilevers in an M-shape. The use of such a design allows for two resonant frequencies at about 1.29 Hz and 1.78 kHz, while the design occupies an area of 2.9 mm × 1 mm. The power output of that work is not reported.

While at the macro scale the literature seems to use cantilevers in order to reach wider bandwidths, at the micro scale, works are exploring variations of existing shapes and new shapes to overcome the limitations of the cantilever in order to increase the performances of vibrational energy harvesters [23,27]. These devices have dimensions ranging from a few millimeters to a few centimeters. Their operating frequency ranges from few hertz to several kilohertz, and their output power varies from a few nanowatts to a few microwatts. 

This paper presents a new architecture for a PEVH design named herein the squared daisy. This structure is demonstrated via the PiezoMUMPS microfabrication process by Memscap. It allows for a structure that is insensitive to process variation and can operate over a relatively wide bandwidth and over different modes. 

The paper is structured as follows: The operating principle of the squared daisy PEVH along with the finite element method (FEM) simulation results are detailed in Section 2. This is followed in Section 3 by the presentation of the fabrication process used for the realization of these devices and the parameters that were selected for fabricated devices. Thereafter, in Section 4, the measurement results are presented, including the characterization of the devices’ variability, resonant frequency, frequency response, and mode shape. Section 5 discusses the devices and compares their performance with other works. Finally, conclusions are presented.

## 2. Materials and Methods

**Figure 1:**
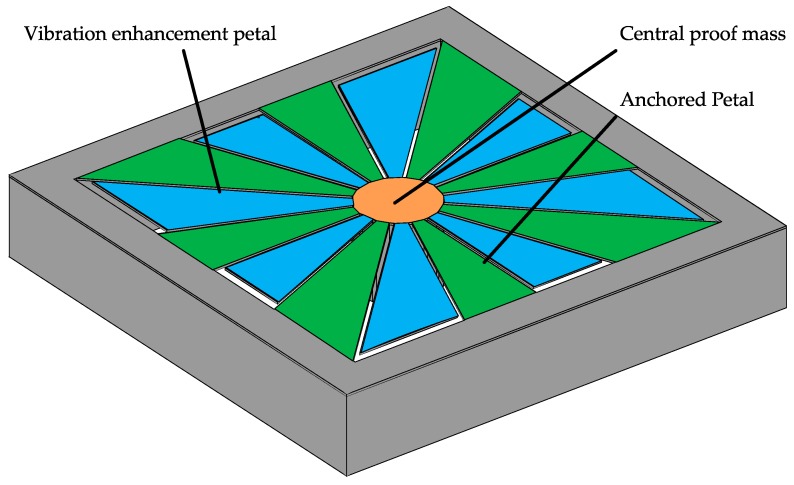
Visualization of the squared daisy design and identification of the main components of the daisy design: A central mass (**orange**) suspended by a predefined number of supporting beams (**green**) and the vibration enhancement petals attached to the central mass (**blue**).

This paper introduces the squared daisy (SD) as a new geometry for the realization of MEMS energy harvesters. This structure aims to be a solution to improve the operating frequency range of MEMS energy harvesters. The visualization of such a structure is presented in Figure 1.

### 2.1. Operating Principle of the Squared Daisy

**Figure 2:**
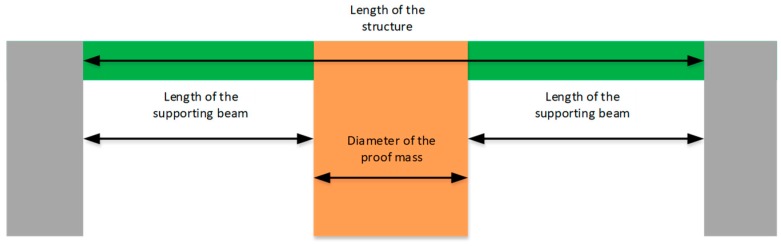
Visualization of a cross-section of the anchored structure.

The SD structure can be simplified into two separate designs: A central proof mass (in orange in Figure 1) suspended by a predefined number of supporting beams (in green in Figure 1) and cantilevers attached to that central mass (in blue in Figure 1). A cross-section is illustrated in Figure 2. The central proof mass is suspended by a predefined number of supporting beams. The fundamental resonant frequency of the structure in Figure 2 can be expressed as a function of *E*, the modulus of elasticity of the material, *I* is the area moment of inertia for that structure, a function of its shape, *Rm* is the radius of the proof mass, *H* is the height of the proof mass, the density of the material *ρ* and *L* is the length of the structure [14]. It is given by:

(1) $$f_{o}=\left(\frac{1}{2\pi }\right)\times \sqrt{\frac{48\times E\times I}{2\times \pi \times Rm\times \rho \times H\times L}}$$

As governed by (1), the greater the force, the lower the resonant frequency. The values of *H* and *a* are fixed, therefore increasing the value of *Rm* should be favorable to reduce the resonant frequency of the device by increasing the force applied to the device. However, increasing the value of *Rm* reduces the effective length of the device, *L_eff_*. This parameter is used to represent the reduction of the length of the supporting cantilevers due to the proof mass. The value of *L_eff_* can be expressed in the function of the total length of the design, *Rm* and a scaling factor *Sc*. The expression of *L_eff_* is given by:

(2) $$L_{eff}=L-\left(2\times Rm\right)\times Sc$$

By combining (1) and (2), the resonant frequency of the design can be shown to be given by:

(3) $$f_{0}=\left(\frac{1}{2\pi }\right)\times \sqrt{\frac{48\times E\times I}{\left(2\times \pi \times Rm\times \rho \times H\right)\times \left(L-\left(2\times Rm\right)\times Sc\right)}}$$

Three main factors have an influence on the resonant frequency of the device: The total length of the device, the radius of the proof mass and the length of the arms. These parameters are linked by the fact that the size of the design is equal to two times the length of the arms and two times the radius of the central mass. While this basic model gives an understanding of the influence of modifying the value of *Rm*, it should be noted that it does not take into account the shapes of the cantilevers supporting the central mass and the fact that not all sides of the proof mass are supported. 

**Figure 3:**
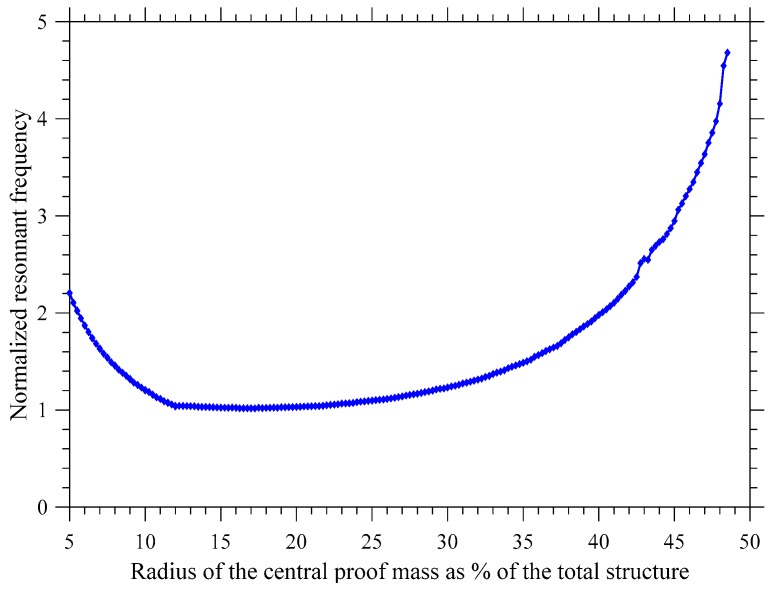
Simulation of the influence of the size of the proof mass on the devices.

To allow for a more complete representation of the behavior of the system, finite element modeling (FEM) of the structure has been carried out using the COMSOL Multiphysics software. The result in terms of the resonant frequency of such a system when modifying the radius of the proof mass and keeping the length of the structure constant is illustrated in Figure 3. To provide a general case, the resonant frequency has been normalized to its lowest value and the proof mass radius, *Rm*, is expressed as the percentage of the total length of the structure. 

It should be noted that if *Rm* is below 5% of the length of the structure then the behavior of this structure changes and is closer to a clamped-clamped cantilever. If *Rm* is equal to 50% of the length of the structure, then the diameter of the proof mass is equal to the length of the structure, and no supporting beams remain.

Two main factors influence the resonant frequency of the system: The longer the supporting beams, the lower the frequency. However, an increase of the length of the arms also implies, for a given total length, a reduction in the radius of the proof mass and therefore an increase of the resonant frequency. It can be seen in Figure 3 that these two effects work in an opposite fashion creating a region where the influence of these two effects counteracts the other. That region is seen in Figure 3 where it can be observed that a change in radius of the proof mass between 10% to 23% of the structure length has little influence on the resonant frequency of the system. This region can be leveraged to reduce the sensitivity of the structure to process variations during device fabrication.

### 2.2. Vibration Enhancement Petals

For the vibration enhancement petals attached to the central mass, these can be modelled as cantilevers. As such, the undamped resonant frequency in the case of a rectangular cantilever can be expressed as a function of a factor used to represent the vibration mode *k_i_*, the length of the cantilever *L*, and the cross-sectional area *A* [8] such that:

(4) $$f_{i}=\frac{k_{i}{}^{2}}{2\pi \times L^{2}}\times \sqrt{\frac{E\times I}{\rho \times A}}$$

In the case of a rectangular beam of sufficient length such that the width of the cantilever can be neglected, the fundamental resonant frequency can be expressed as a function of its length as:

(5) $$f_{i}=\frac{1.875^{2}}{2\pi }\times \sqrt{\frac{E\times I}{\rho \times L^{3}}}$$

It should be noted that in this case, while all the vibration enhancement petals are situated around the central proof mass, they do not have the same properties, in terms of length and width at the end, it is therefore expected that these small variations will result in small resonant frequency variations that will allow for a wider overall bandwidth.

It should be noted that in a trapezoidal beam such as that of the petals, the cross-sectional area is not a constant over the length of the cantilever as the width of the cantilever is equal to *wb1* at one end and *wb2* at the other. As such, the expression of the aforementioned expressions can only give a rough estimate of the fundamental mode. 

An analytical model of the SD structure is beyond the scope of this work. More advanced material on the analytical modeling of cantilevers and more complex structures can be found in [7,8,14,22,23].

These cantilevers are introduced by vibration enhancement petals (VEP), as their presence allows the addition of their resonant frequency to the global resonant behavior of the structure. Furthermore, by having the VEP supported from the central mass, the structure exhibits different properties in terms of frequency response and fundamental resonant mode, as will be discussed later.

### 2.3. Resilience to Process Variation 

Process variation is an issue in microfabrication. In this particular case, the PiezoMUMPS process allows for an edge bias of up to 50 µm in the trenches below the silicon structure of the SD [28]. This can vary the length of the suspended structure and affect resonant behavior. Accordingly, in order to limit the influence of such process variation, two main strategies have been used. The first strategy used was to anchor the design on all sides such that the surrounding silicon structure overhangs the underlying trench at the perimeter of the structure, as demonstrated in an ultrasonic transducer demonstrated in [29]. However, while this strategy reduces the influence of the process variation on the anchoring at the perimeter of the structure, it will have no influence on process variation touching the central proof mass. This proof mass is a key component in this design to reduce the resonant frequency. Therefore, a variation of the proof mass radius by up to 50 µm would render such a design difficult to fabricate reliably. However as stated previously, in this design, a region in which the variation of the radius of the proof mass has a negligible influence on the resonant frequency of the design exists, as seen in Figure 3. This increases the resilience of the device to process variations of the SD design and ensures that the resonant frequency does not vary significantly. 

## 3. Fabrication

In the previous section, the operating principle of the squared daisy has been overviewed. The fabrication process used to realize and validate the operation of these devices is detailed, along with the device parameters that have been selected.

**Figure 4:**
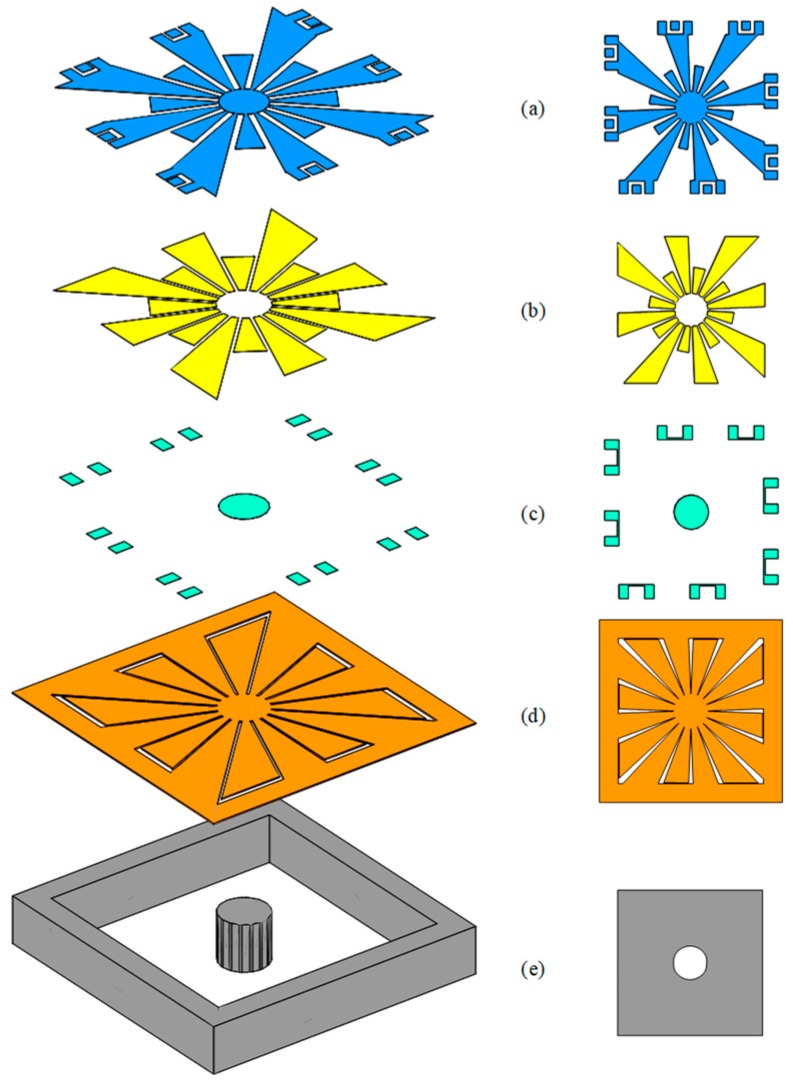
Illustration of the PiezoMUMPs process layers that compose the square daisy: (**a**) Metal aluminum pads, (**b**) piezoelectric AlN, (**c**) silicon dioxide insulator, (**d**) silicon device layer, and (**e**) trenched silicon substrate (left: Isometric view, right: Top view).

The layout for the fabrication of these devices has been scripted using the SKILL language using the layout tool Cadence Virtuoso. SKILL was used to describe one parameterized cells (PCell) that contained the layout of the SD design. This PCell describes the layout of the devices, and allows for easy scaling of the device and variation of its parameters. The different layers of one variation of the SD design are shown in Figure 4, outlining each of the masks of the process. In that design all the dimensions are controlled via dimensions that are parameterized and each petal can be defined as an anchored petal or as a vibration enhancement petal. 

The fabrications of these prototypes were carried-out using the PiezoMUMPs process from MEMSCAP. This process is a piezoelectric-based MEMS process that provides cost-effective access to MEMS prototyping. This process has been used and described for the creation of several resonators and energy harvesters [20,21,25,30,31]. The fabrication process includes five masks and is carried out on an N-type double-side polished silicon-on-insulator (SOI) wafer. The 10 µm-thick silicon layer (Figure 4d) is doped to increase its conductivity. Then, an insulating 0.2 µm-thick layer of silicon dioxide is grown and patterned on the SOI wafer (Figure 4c). A 0.5 µm-thick piezoelectric layer of aluminum nitride (AlN) is then deposited and patterned (Figure 4b). Then, a layer of metal is deposited, this layer consists of a stack of 0.02 µm-thick chromium (Cr) and of 1 µm-thick aluminum (Al) (Figure 4a). The silicon device layer is then patterned (Figure 4d). Lastly, the 400 µm substrate is etched from the backside to form the trench below the structure and free the proof mass to enable the structure to vibrate (Figure 4e). Further details pertaining to the fabrication process can be found in [28]. It is worth noting that this process allows for the use of the suspended substrate to be used as a proof mass. Upon reception of the device from the foundry, no post-processing step was applied to the devices.

In order to validate the use of the SD design and the validity of the frequency enhancement petals, three different design variations were created. The parameters used to create these structures are presented in Table 1. The *size* parameter of the design represents the side of the square in which the design fits, however, this does not take into account the space needed to place the bond pads. The parameter *radius of the proof mass* represents the radius of the circle that is used to create the proof mass. The *separation on the VEP* parameter is used to determine the distance between the end of the vibration enhancement petals and the substrate. The *radius of the circle of deposited piezo* parameter is used to determine the area covered in piezo material on the VEPs. The *petals used as anchors* and *petals used as a VEP* parameter are complementary (each petal is either used as an anchor or a VEP) and are used to determine the anchoring of the structure. The use of these parameters allowed the realization of the three variants using a single PCell.

**Figure 5:**
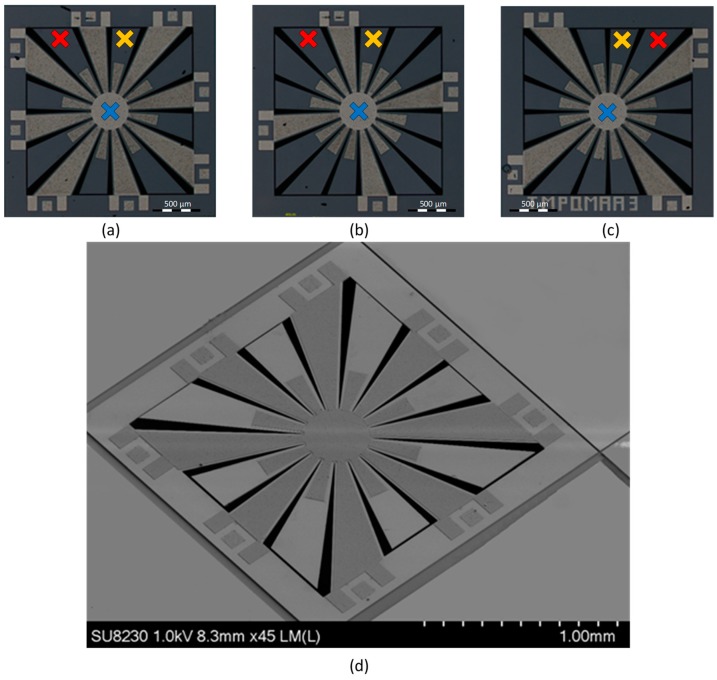
Presentation of the 3 fabricated designs the variant 1 in (**a**) has 8 anchored petals and 8 vibrations enhancement petals, while both variant 2 in (**b**) and variant 3 in (**c**) have 4 anchored petals and 12 vibration enhancement petals. An isometric view of variant 1 is provided in (**d**).

The only difference between these three variants is the way the petals are used. In the first variant, eight petals are used as anchors while eight are used as VEP. In the second and third variant, it was chosen to use only four petals as anchors while the 12 others are used as vibration enhancement petals. Pictures of the realized structures are shown in Figure 5. Fifteen copies of each of these devices were received. In that figure, the measurements points “Center”, “VEP1” and “VEP2”, respectfully in blue, red and orange are clearly identified for the variants and will be referred to in the measurements presented in Section 4. For the isometric view provided for variant 1, it is not possible to see the central proof mass due to the too small opening between the petals at the center of the structure.

## 4. Measurement Results 

In this section, the characterization of the performance of the fabricated SD designs is presented. This includes the measurement of their resonant behavior, the characterization of their frequency response in terms of power output, and the visualization of their mode shapes.

### 4.1. Characterization Using a Vector Network Analyzer

To characterize the resonant frequency of the designs in terms of frequency response, a vector network analyzer (VNA) type E5061B from Keysight was used with a probe station. A hole is present below the devices in order to allow the displacement of the central proof mass. By measuring the S parameters of these devices (S21 and S12), this test allowed a quick characterization of the variants. The resonant frequency of all the devices were recorded and stored, for each variant the median, average and the standard deviation of the resonant frequency was calculated. For variant 3, the extraction of the first resonant mode with the VNA was not possible due to limitation of the test setup. However, the extraction of the second mode at 7.3 kHz was possible and should be indicative of the variation of the 1st mode. The fundamental mode of variant 3 was extracted using a vibrometer, as discussed in Section 4.2. Table 2 presents a summary of these measurements. It can be noted that the good agreement of the simulations and the measurements. The maximum standard deviations in terms of resonant frequency is below 3.11% over the 15 devices measured for each variant. These results validate both of the approaches used in order to reduce the sensitivity of the structure to process variations and also validate the FEM simulations in terms of resonant frequencies.

### 4.2. Characterization Using a Vibrometer

**Figure 6:**
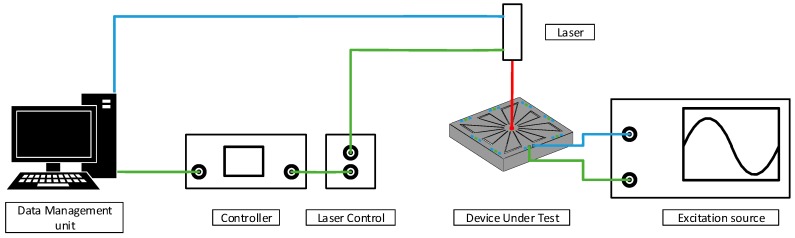
Schematic of the vibrometer test bench.

A vibrometer is capable to make non-contact vibration measurements of a surface. It is possible to use it to acquire the frequency response in terms of velocity of a point. This frequency response allows the identification of the resonant mode. Furthermore, the use of a vibrometer allows the visualization of the mode shape of the device, via the measure of the speed of 2500 points at regular intervals over the area covered by the MEMS device. A data management system from Polytec is used, the vibrometer controller used is the OFV-2570 from Polytec and the laser unit is the OFV-534 from Polytec. This equipment setup is presented in Figure 6. To obtain these results, the excitation of the PVEH is provided using a function generator type 33,250A from Agilent. This excitation source is used to generate a sweep in frequency from 1 kHz to 20 kHz at a constant amplitude. 

**Figure 7:**
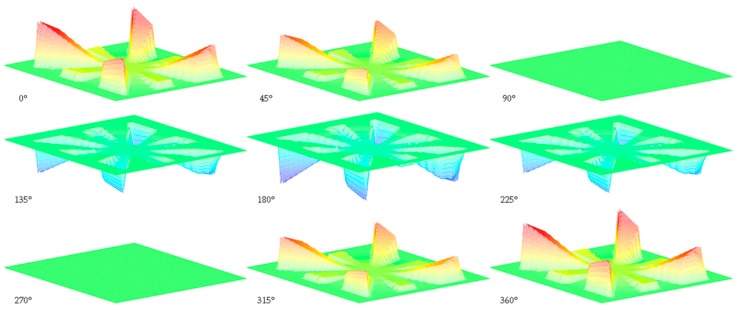
Representation of the 1st mode shape of variant 1 as a function of the phase.

The vibrometer is used to acquire the displacement velocity of each point as a function of the frequency. Using a positioning controller type Corvus Evo from PI and 2 positioners type VT-80 Linear Stage from PI. Using this equipment, 2500 points linearly spaced in a 50 × 50 grid around the design can be defined. Using this grid, the movement of the structure can be reconstructed at a defined frequency, as the exact coordinates where the points are measured are known. An example of the representation of the mode shape for variant 1 at its first resonant frequency is shown in Figure 7. 

To realize this measurement, variant 1 was exposed to a sinusoidal signal of amplitude 10 V at a frequency of 10.86 kHz. At this frequency, the resonant mode of the variant 1 is a combination of three vibration patterns. The first is caused by petals number 2, 6, 10 and 14 and presents the maximal displacement. The second is caused by petals number 4, 8, 12 and 16, with an amplitude that is lower than the first one. These two resonant patterns are different as the dimensions of these petals are different as shown in Figure 5, leading to different resonant frequencies for both. Finally, the third vibration pattern is one of the central proof mass with an amplitude that is the lowest but that also causes the displacement of the petals number 1, 3, 5, 7, 9, 11, 13 and 15. 

**Figure 8:**
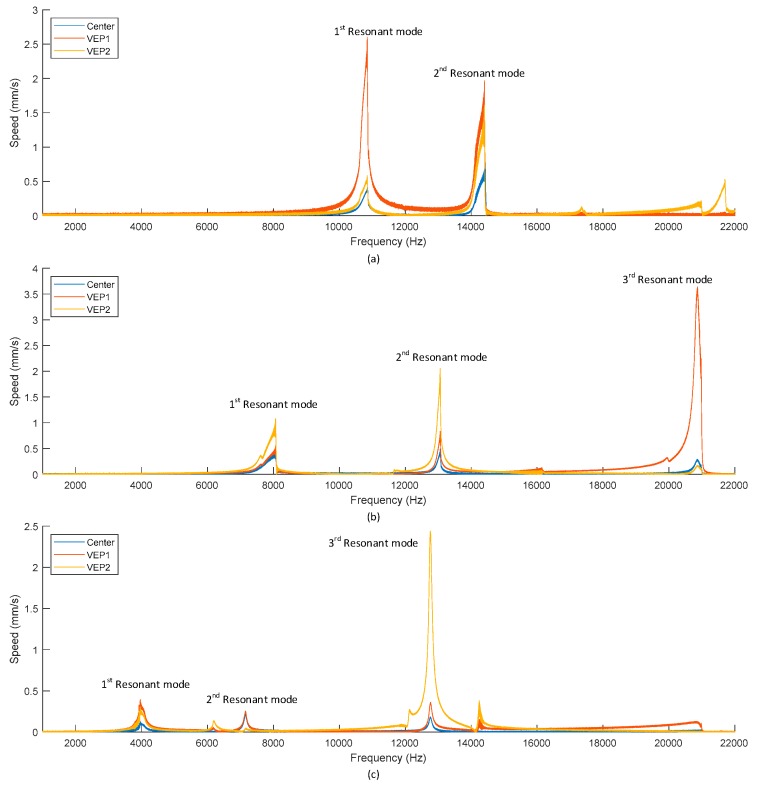
Velocity response of the variant 1 in (**a**), variant 2 in (**b**) and variant 3 in (**c**) from 1 kHz to 22 kHz.

This analysis is consistent with the data presented in Figure 8, which shows the velocity frequency response of three different locations on the device. For each of the variants, the frequency response in terms of velocity from 1 kHz to 22 kHz has been characterized at the Center, VEP1 and VEP2 points on the device, while it is subjected to a sinusoidal electrical excitation signal of 20 V amplitude. The positions of the measured points on the device are outlined in Figure 5. The test setup used is the same as described previously. These results are presented in Figure 8. It can be observed that each design possesses several resonant modes between 1 kHz and 22 kHz, in addition, several of these modes present non-linear properties. Concerning the amplitude of these modes, it is noticeable that the amplitude of the velocity of points VEP1 and VEP2 is usually higher than the one of the center. Furthermore, some modes present a higher velocity. These modes are expected to achieve a higher power output. The characteristics of the resonant modes of the central proof mass when exposed to a sinusoidal excitation signal of 20 V amplitude are summarized in Table 3. Some modes have been omitted from this table as they are not present in all of the three measured points or because they only are relatively weak, becoming apparent after a higher level of excitation has been applied. FEM simulation results of the resonant frequency have been included to highlight the good agreement between measurement and simulation. 

**Figure 9:**
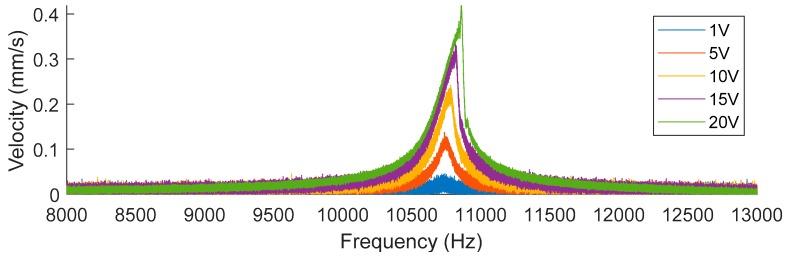
Frequency response around first resonant mode of variant 1 in terms of the velocity of the center point as a function of the amplitude of the excitation signal provided.

It should be noted that for the nonlinear modes, the start frequency, the resonant frequency, and stop frequency will vary as a function of the amplitude of the excitation [32], furthermore this non-linear behavior will only occur after a sufficient level of excitation has been reached, as can be observed in Figure 9 for the first resonant mode of variant 1. In this figure, the displacement velocity of the central point is represented from 8 kHz to 13 kHz for several levels of excitation. If the amplitude of the sinusoidal excitation signal is below 5 V, then the behavior of the device is linear. However, if the amplitude is higher than 10 V then the behavior will start to appear as non-linear. This behavior is consistent with the behavior of duffing oscillators such as described in [33].

These measurements, combined with the frequency response of the device validates the hypothesis about the use of several vibration enhancement petals. The petals used as VEP allow increasing the amplitude of the frequency response of the device in addition to offering additional resonant modes. Furthermore, these petals present resonances at the same frequency as the central proof mass, this behavior should allow an improvement of the power output of the device at these frequencies.

### 4.3. Characterization Using a Shaker

**Figure 10:**
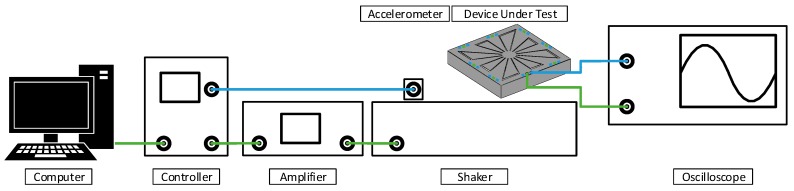
Presentation of the mechanical test bench for MEMS devices.

The performance in terms of output power and voltage output was characterized with a shaker type 4809 from Brüel&Kjær. This shaker is controlled by a Comet unit from Brüel&Kjær, a power amplifier type 2718 Brüel&Kjær, used with an accelerometer type 4517 from Brüel&Kjær in order to allow for proper control of the excitation signal in terms of acceleration and frequency. Regarding the data acquisition, an oscilloscope type DSO-X 3034A from Keysight is used. Once recorded, the data is submitted to an FFT algorithm to extract the amplitude of the harvested voltage. An illustration of the test setup is provided in Figure 10. However, due to frequency range operation limitations, the current test setup does not allow to characterize the performance of variant 1. 

**Figure 11:**
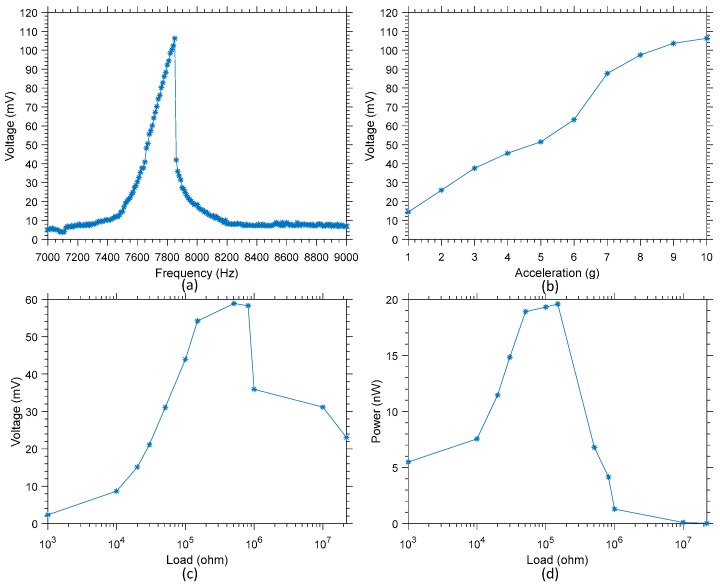
(**a**) Frequency response of variant 2 when subjected to an acceleration of 10 g between 7 and 9 kHz, (**b**) variation of the voltage output amplitude as a function of the input acceleration, (**c**) variation of the voltage output as a function of the load at resonance, and (**d**) and variation of the power output as a function of the load at resonance.

**Figure 12:**
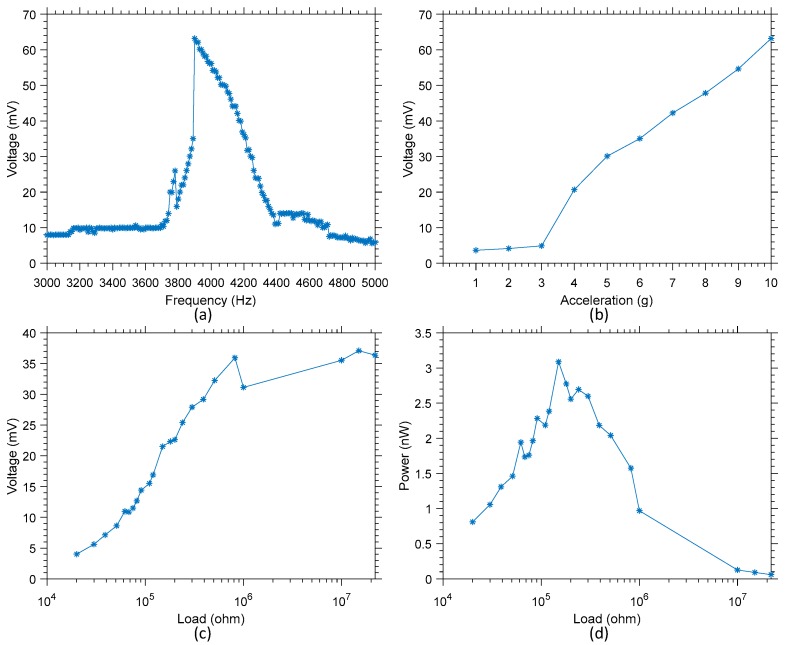
(**a**) Frequency response of D3 when subjected to an acceleration of 10 g between 3 kHz and 5 kHz, (**b**) variation of the voltage output amplitude as a function of the input acceleration, (**c**) variation of the voltage output as a function of the load at resonance, and (**d**) variation of the power output as a function of the load at resonance.

The frequency response in open circuit of variants 2 and 3, when subjected to an acceleration of 10 g, has been characterized around resonant mode 1, and is presented in Figure 11a and Figure 12a, respectively. This data allowed the confirmation of the resonant frequency of 7.85 kHz for variant 2 where a maximum of 106 mV (amplitude) is harvested, and 3.9 kHz for variant 3 where a maximum of 64 mV (amplitude) is harvested. These frequencies will be the one used to further characterize the performances of these harvesters.

In Figure 11b and Figure 12b, the variation of the open circuit voltage output is presented as a function of the acceleration. The apparition of non-linearity in the voltage output can be noted if the device is subjected to an acceleration greater than 6 g and 3 g, respectively for variant 2 and 3. This behavior is consistent with the data presented in Figure 9 for variant 1, and of the behavior of duffing oscillators [33]. It should be noted that accelerations of 6 g at 7.85 kHz or of 3 g at 3.9 kHz correspond in both variants to a displacement speed of about 1.2 mm/s. However, this corresponds to a displacement of about 0.15 µm in variant 2, while it corresponds to a displacement of about 0.30 µm in variant 3.

Finally, Figure 11c,d present the output voltage and power as a function of the load for variant 2, while Figure 12c,d present the output voltage and power as a function of the load for variant 3. For variant 2, a maximum of 20 nW is harvested, while for variant 3 a maximum of 3.1 nW of power is harvested when excited at their respective resonant frequency. It is worth noting that both these maximum were reached with a load of 150 kΩ for both devices. The difference in terms of maximum output power between these two variants is consistent with the velocity of the displacement measured and presented in Figure 8. Indeed, when excited at resonance with a similar signal amplitude, the velocity of variant 2 is much higher than that of variant 3. A higher velocity results in more displacement over a given time, and in turn more strain variation over the piezo material, resulting in higher output power.

For each of these structures, their multimode ability has been demonstrated, a visualization of the main resonant modes of the structures has been provided. In addition, their frequency response in terms of output voltage and power has been quantified. Furthermore, if a sufficient level of excitation is reached, then non-linear characteristics can be observed. This observation has been confirmed for both types of excitation: Electrical or mechanical. The low sensitivity to process the variation in terms of variation of the resonant frequency of the SD has been characterized for each variant. Concerning the reliability of the devices, these tests have occurred over a period of one year, and no device has failed during excitations that were applied. Due to their small size and high resonant frequencies, it is possible to expose them to high accelerations (~10 g) without risking their destruction.

## 5. Discussion

This section presents a comparison of the proposed devices with devices found in the literature. The performance of the SD design and of others are summarized and compared.

As stated in the introduction, several other designs have been proposed to overcome the limitation in terms of the operational bandwidth of cantilevers [1,13,34,35,36]. The performance and main characteristics of these works are summarized in Table 4. This table allows a quick overview of the performance of several devices intended to be used as MEMS PVEH energy harvesters.

In order to compare the performances of these devices, one of the most common figures of merit (FOM) has been considered. The FOM expresses the maximum power output of the device in function of the volume used by the harvester and the intensity of the acceleration the device was subjected to, it is therefore expressed in nW·mm^−3^·g^−2^. However, it should be noted that this figure of merit has several limitations, as it is unable to express the difference between an acceleration of 10 g at 100 Hz versus an acceleration of 10 g at 10 kHz, as the first one corresponds to a displacement of 156 µm while the second one corresponds to a displacement of 0.156 µm. This difference is important as in the case of piezoelectric energy harvester, the energy is created by straining of the piezo material, and therefore the displacement magnitude has an impact on the output power. Furthermore, this figure of merit does not consider the operating bandwidth of the devices, which is an important metric of PVEH devices.

Accordingly, the FOM performance of the presented devices falls behind, as they exhibit high resonant frequencies and thus smaller displacement for a given acceleration. The performance of variant 2 is better according to this FOM than variant 3 while the operating frequency of variant 2 is about two times higher than the one of variant 3. Small devices with non-linearity and multimode capability [34] or bigger devices [36] are able to respond to very low accelerations, at low frequency, resulting in a large displacement, increased output power, and better FOM. Future work will be based on increasing the surface of piezo material where strain is present to maximize the power output of the SD device. Additional effort will be made to augment the number of resonant modes of the structures and their non-linearity. Scaling this device up in order to reduce the resonant frequency, reduces the acceleration needed to achieve sufficient displacement, and increasing the maximum power output will allow the devices to better compare with other works using the FOM. 

The current dimensions, operating frequency range, response to very small displacement, and their ability to exploit several nonlinear resonances yielding operational bandwidth versatility, renders these prototyped harvester suitable for use in acoustic energy harvesting applications. 

Moreover, a scale up the devices should allow the placement of proof masses on the vibration enhancement petals, in order to reduce their resonant frequency further and add more resonances to the system such as the ones observed in [3]. 

According to FEM simulations, if the size of the design is set to 4200 µm to occupy a full area of a block of the PiezoMUMPS process, the resonance frequency can be reduced. The volume is thus increased to 7.056 mm^3^, and the operating frequency of variant 2 becomes 1.1 kHz, and that of variant 3 becomes 550 Hz. At these frequencies, the acceleration needed to obtain a similar displacement to the one presented in this table is of 0.2 g for variants 2 and 3. These values are consistent with the ones presented in [13,32,34], and indicate the potential of the design towards significantly increasing its FOM, when scaled to operate at frequencies similar to those of other works. In addition, note that this scale up does not assume that additional proof masses that could be added to a larger structure further reducing the resonant frequency.

## 6. Conclusions

This paper presented a new proof of concept architecture for the realization of MEMS PVEH: The squared daisy. This architecture allows for the operations of a nonlinear multi-mode energy harvester. In addition, this harvester is relatively immune to process variations due to its unique architecture. The concept of vibration enhancement petals was introduced and three variants of the designs were fabricated. The performance in terms of frequency response and variation of the power output as a function of the acceleration amplitude was given, and the visualization of the mode shape of the device, when excited at one of its resonant frequency, was provided. Future work will investigate scaling up of these devices in order to improve their power output and reduce their frequency range to allow for their use in energy harvesting applications.

## Figures and Tables

**Table 1:** Overview of the parameters for each of the three variations of the SD design.

	Variant 1	Variant 2	Variant 3
Size of the design (µm)	1700	1700	1700
Radius of the proof mass, *Rm* (µm)	200	200	200
Separation on the VEP (µm)	10	10	10
Radius of the circle of deposited piezo material on VEP(µm)	500	500	500
Petals used as anchors	1 3 5 7 9 11 13 15	1 5 9 13	3 7 11 15
Petals used as a VEP	2 4 6 8 10 12 16	2 3 4 6 7 8 10 11 12 14 15 16	1 2 4 5 6 8 9 10 12 13 14 16

**Table 2:** Summary of the resonant frequency of the three variations of the squared daisy (SD) design.

	Variant 1	Variant 2	Variant 3 *
Simulation (Hz)	10,600	8100	7300
Median (Hz)	10,680	8120	7286
Mean (Hz)	10,637	8141	7308
Standard deviation (Hz)	129	196	227
Standard deviation (%)	1.21	2.41	3.11

* Second mode.

**Table 3:** Summary of the characteristics of the resonances for the three variants at 20 V excitation.

		Variant 1	Variant 2	Variant 3
Resonant Mode 1	Start Frequency (Hz)	10,696	7466	3906
	Measured | Simulated Resonant Frequency (Hz)	10,860 | 10,800	7902 | 8100	3971 | 4050
	Stop Frequency (Hz)	10,887	7922	4138
	Linearity	Non-linear	Non-linear	Non-linear
Resonant Mode 2	Start Frequency (Hz)	14,216	12,839	7098
	Measured | Simulated Resonant Frequency (Hz)	14,485 | 15,000	12,941 | 13,100	7166 | 7243
	Stop Frequency (Hz)	14,507	12,973	7243
	Linearity	Non-linear	Non-linear	Linear
Resonant Mode 3	Start Frequency (Hz)	--	20,744	12,688
	Measured | Simulated Resonant Frequency (Hz)	--	20,871 | 20,987	12,757 | 12,833
	Stop Frequency (Hz)	--	20,987	12,833
	Linearity	--	Non-linear	Linear

**Table 4:** Performances of the designs and comparison with the state of the art.

	Variant 2	Variant 3	[1]	[13]	[34]	[35]	[36]
**Volume (mm^3^)**	1.156	1.156	57.6	25.6	14.4	112	6500
**Piezo material**	AlN	AlN	AlN	AlN	AlN	AlN	PZT
**Operating bandwidth (Hz)**	7466–7922	3906–4138	730–1000	190	85–95	859.9–924.5	387–398
**Linearity**	Non-linear	Non-linear	Non-linear	Linear	Non-linear	Non-linear	Linear
**Acceleration (g)**	10	10	1	15	0,2	2	0.05
**Output Voltage (mV)**	54.2	21.52	770.3	130	1000	4433	1000
**Load**	150 kΩ	150 kΩ	70 kΩ	1 MΩ	1 MΩ	100 kΩ	1 MΩ
**Output Power (nW)**	20	3.1	1730	169	136	82240	52900
**FOM nW·mm^−3^·g^−2^**	0.17	0.3	30.03	0.03	236.11	183.82	3255

## References

[1] Jia Y., Du S., Seshia A.A. (2016). Twenty-Eight Orders of Parametric Resonance in a Microelectromechanical Device for Multi-band Vibration Energy Harvesting. Sci. Rep..

[2] Toyabur R.M., Salauddin M., Park J.Y. (2017). Design and experiment of piezoelectric multimodal energy harvester for low frequency vibration. Ceram. Int..

[3] Iannacci J., Serra E., Sordo G., Bonaldi M., Borrielli A., Schmid U., Bittner A., Schneider M., Kuenzig T., Schrag G. (2018). MEMS-based multi-modal vibration energy harvesters for ultra-low power autonomous remote and distributed sensing. Microsyst. Technol..

[4] Nabavi S., Zhang L. (2019). Frequency Tuning and Efficiency Improvement of Piezoelectric MEMS Vibration Energy Harvesters. J. Microelectromech. Syst..

[5] Vyas A., Staaf H., Rusu C., Ebefors T., Liljeholm J., Smith A.D., Lundgren P., Enoksson P. (2018). A Micromachined Coupled-Cantilever for Piezoelectric Energy Harvesters. Micromachines.

[6] Brini O., Deslandes D., Nabki F. (2019). A System-Level Methodology for the Design of Reliable Low-Power Wireless Sensor Networks. Sensors.

[7] Erturk A., Inman D.J. (2011). Front Matter. Piezoelectric Energy Harvesting.

[8] Priya S., Inman D.J. (2009). Energy Harvesting Technologies.

[9] Podder P., Constantinou P., Mallick D., Amann A., Roy S. (2017). Magnetic Tuning of Nonlinear MEMS Electromagnetic Vibration Energy Harvester. J. Microelectromech. Syst..

[10] Markowski P. (2014). Thermoelectric energy harvester fabricated in thick-film/LTCC technology. Microelectron. Int..

[11] Fowler A.G., Moheimani S.O.R. (2016). A 4-DOF MEMS Energy Harvester Using Ultrasonic Excitation. IEEE Sens. J..

[12] Beeby S.P., Tudor M.J., White N.M. (2006). Energy harvesting vibration sources for microsystems applications. Meas. Sci. Technol..

[13] Iannacci J., Sordo G., Serra E., Schmid U. A novel MEMS-based piezoelectric multi-modal vibration energy harvester concept to power autonomous remote sensing nodes for Internet of Things (IoT) applications. Proceedings of the 2015 IEEE SENSORS.

[14] Brand O., Dufour I., Heinrich S., Josse F., Fedder G.K., Hierold C., Korvink J.G., Tabata O. (2015). Resonant MEMS: Fundamentals, Implementation, and Application.

[15] Yu H., Zhou J., Deng L., Wen Z. (2014). A vibration-based MEMS piezoelectric energy harvester and power conditioning circuit. Sensors.

[16] Chew Z., Li L. (2010). Design and characterisation of a piezoelectric scavenging device with multiple resonant frequencies. Sens. Actuators A Phys..

[17] Li S., Crovetto A., Peng Z., Zhang A., Hansen O., Wang M., Li X., Wang F. (2016). Bi-resonant structure with piezoelectric PVDF films for energy harvesting from random vibration sources at low frequency. Sens. Actuators A Phys..

[18] Seddik B.A., Despesse G., Boisseau S., Defay E. (2012). Strategies for Wideband Mechanical Energy Harvester.

[19] Zhu D., Beeby S.P. Scaling effects for piezoelectric energy harvesters. Proceedings of the Smart Sensors, Actuators, and MEMS VII; and Cyber Physical Systems.

[20] Alameh A., Gratuze M., Elsayed M., Nabki F. (2018). Effects of Proof Mass Geometry on Piezoelectric Vibration Energy Harvesters. Sensors.

[21] Alameh A.H., Gratuze M., Robichaud A., Nabki F. Study and Design of MEMS Cross-Shaped Piezoelectric Vibration Energy Harvesters. Proceedings of the 2018 25th IEEE International Conference on Electronics, Circuits and Systems (ICECS).

[22] Dompierre A., Vengallatore S., Fréchette L.G., Vengallatore S., Fréchette L.G. (2013). Piezoelectric Vibration Energy Harvesters Modeling, Design, Limits, and Benchmarking. Energy Harvesting with Functional Materials and Microsystems.

[23] Beeby S.P. (2015). Energy Harvesting Devices. Resonant MEMS.

[24] Dhote S., Yang Z., Zu J. (2018). Modeling and experimental parametric study of a tri-leg compliant orthoplanar spring based multi-mode piezoelectric energy harvester. Mech. Syst. Signal Process..

[25] Yaghootkar B., Azimi S., Bahreyni B. Wideband piezoelectric mems vibration sensor. Proceedings of the 2016 IEEE SENSORS.

[26] Hoffmann D., Bechtold T., Hohlfeld D. Design optimization of MEMS piezoelectric energy harvester. Proceedings of the 2016 17th International Conference on Thermal, Mechanical and Multi-Physics Simulation and Experiments in Microelectronics and Microsystems (EuroSimE).

[27] Zhu D., Tudor M.J., Beeby S.P. (2009). Strategies for increasing the operating frequency range of vibration energy harvesters: A review. Meas. Sci. Technol..

[28] Cowen A., Hames G., Glukh K., Hardy B. (2014). PiezoMUMPs Design Handbook.

[29] Robichaud A., Deslandes D., Cicek P., Nabki F. (2018). A Novel Topology for Process Variation-Tolerant Piezoelectric Micromachined Ultrasonic Transducers. J. Microelectromech. Syst..

[30] Pons-Nin J., Gorreta S., Dominguez M., Blokhina E., O’Connell D., Feely O. Design and test of resonators using PiezoMUMPS technology. Proceedings of the 2014 Symposium on Design, Test, Integration and Packaging of MEMS/MOEMS (DTIP).

[31] Elsayed M.Y., Nabki F. (2017). Piezoelectric Bulk Mode Disk Resonator Post-Processed for Enhanced Quality Factor Performance. J. Microelectromech. Syst..

[32] Nabavi S., Zhang L. (2019). Nonlinear Multi-mode Wideband Piezoelectric MEMS Vibration Energy Harvester. IEEE Sens. J..

[33] Kovacic I., Brennan M.J. (2011). The Duffing Equation: Nonlinear Oscillators and Their Behaviour.

[34] Rezaeisaray M., Gowini M.E., Sameoto D., Raboud D., Moussa W. (2015). Low frequency piezoelectric energy harvesting at multi vibration mode shapes. Sens. Actuators A Phys..

[35] Wang N., Sun C., Siow L.Y., Ji H., Chang P., Zhang Q., Gu Y. AlN wideband energy harvesters with wafer-level vacuum packaging utilizing three-wafer bonding. Proceedings of the 2017 IEEE 30th International Conference on Micro Electro Mechanical Systems (MEMS).

[36] Aktakka E.E., Najafi K. Three-axis piezoelectric vibration energy harvester. Proceedings of the 2015 28th IEEE International Conference on Micro Electro Mechanical Systems (MEMS).

